# Frequency, distribution, and prognostic impact of metastatic site in dogs with splenic hemangiosarcoma

**DOI:** 10.1093/jvimsj/aalag027

**Published:** 2026-02-17

**Authors:** Paola Valenti, Barbara Bacci, Chiara Catalucci, Elisabetta Treggiari, Marco Luigi Bianchi, Giulia Capra, Giancarlo Avallone

**Affiliations:** Clinica Veterinaria Malpensa AniCura, Samarate 21017, Italy; Department of Veterinary Medical Sciences (DIMEVET), University of Bologna, Ozzano dell’Emilia 40064, Italy; Clinica Veterinaria Malpensa AniCura, Samarate 21017, Italy; Oncopets Veterinary Centre, Milan 20142, Italy; DocVet, Nerviano 20014, Italy; Clinica Veterinaria Malpensa AniCura, Samarate 21017, Italy; Department of Veterinary Medical Sciences (DIMEVET), University of Bologna, Ozzano dell’Emilia 40064, Italy

**Keywords:** metastasis, prognosis, stage, splenic endothelial cell neoplasm

## Abstract

**Background:**

Splenic hemangiosarcoma (SHSA) is an aggressive neoplasm of dogs characterized by high metastatic rate and short survival time. Although staging and treatment are well established prognostic factors, the implication of specific metastatic sites remains unclear.

**Hypothesis/Objectives:**

Describe the frequency and distribution of metastatic site at diagnosis in dogs with SHSA and evaluate the potential prognostic role of different metastatic locations.

**Animals:**

Sixty-six dogs with histologically confirmed SHSA.

**Methods:**

Retrospective, multicenter, descriptive study of dogs with SHSA treated by splenectomy. Data collected included demographics, clinical stage, and site of metastasis at diagnosis and at death, staging procedures, histopathology results, treatment protocols, and outcome. Survival analysis was conducted using Kaplan-Meier and Cox proportional hazards models.

**Results:**

At diagnosis, three dogs were stage I (5%), 35 stage II (53%), and 28 stage III (42%). Overall median tumor-specific survival (TSS) was 132 days. Stage III disease and hepatic metastases were associated with significantly decreased survival (*P* < .001). Dogs with liver metastasis that received anthracycline-based chemotherapy had longer survival compared with dogs that received metronomic therapy (255 vs 65 days, *P* = .02). Muscular and pulmonary metastases did not correlate with worse outcomes.

**Conclusions and clinical importance:**

Stage and treatment were confirmed as prognostic factors, with patients in stage III and patients having received surgery alone having a worse prognosis. Although current staging classifies all metastatic disease as stage III, metastatic site may have variable impact on survival and should be considered when devising treatment strategy.

## Introduction

Hemangiosarcoma is a malignant neoplasm of vascular endothelial origin,^1^ accounting for approximately 5%-7% of all malignancies in dogs.^2^ Several breeds, including Golden retriever, Labrador retriever, Boxer, German shepherd, and other large breed dogs are prone to developing splenic hemangiosarcoma (SHSA).^2^

Although hemangiosarcoma can arise in any tissue or organ containing vascular structures, the spleen, heart, liver, retroperitoneum, and skin are the most frequently affected sites.^3^ Metastases typically occur in the liver, mesentery, omentum, and lungs^4–6^ via the hematogenous route or by transcoelomic spread of neoplastic cells.

The spleen is the most common anatomical location of hemangiosarcoma in dogs and the tumor is characterized by a high rate of metastasis at the time of diagnosis and short survival time. One year survival rates for stage II/III disease are typically <20%.^5,7,8^

Clinically, SHSA can be categorized using a 3-tier system^9^ (Table 1): Stage I refers to tumors <5 cm in diameter, confined to the spleen, with no regional lymph node involvement and no evidence of distant metastasis; stage II refers to ruptured splenic tumors or tumors >5 cm in diameter with or without regional lymph node involvement and stage III refers to tumors with distant metastases. Currently, clinical stage and treatment remain the most reliable prognostic indicators, with shorter median survival time (MST) for stage II and III compared with stage I.^8,10,11^ In particular, dogs with macroscopic metastatic disease (ie, stage III), have a worse prognosis and, in patients treated with surgery with or without chemotherapy, reported survival times range between 68 and 136 days.^12–16^

**Table 1:** Clinical TNM staging system for dogs with HSA.^9^

**TNM and stage**	**Description**
**T**	T0 = no evidence of tumor T1 = tumor < 5 cm confined to the primary site T2 = tumor ≥5 cm confined to the primary site or ruptured T3 = tumor involving adjacent structures
**N**	N0 = no lymph node involvement N1 = regional lymph node involvement N2 = distant lymph node involvement
**M**	M0 = no distant metastasis M1 = distant metastasis
**Stages**	I = T0 or T1; N0; M0 II = T1 orT2; N0 or N1; M0 III = T2 or T3; N0, N1 or N2; M1

Other proposed negative prognostic factors, such as high proliferative activity, high tumor grade, anemia, thrombocytopenia, or the need for blood transfusion at the time of diagnosis have been reported inconsistently.^17–19^

Splenectomy combined with chemotherapy is the treatment associated with the longest outcomes: A significant difference in MST has been reported, with a MST of 2 months reported for dogs that received surgery alone and 6-8 months for dogs treated with surgery and adjuvant chemotherapy.^5,7,19^

The most effective chemotherapy protocols are anthracycline (AC)-based, used alone or in combination with other antineoplastic agents.^12,15,16,20,21^ The use of metronomic chemotherapy (MC) also has become a therapeutic option, mainly in the adjuvant setting,^11,14,22^ whereas for advanced stage disease, to improve the clinical outcome, multiagent protocols have been proposed, but comparative studies demonstrating the superiority of one protocol over another are lacking currently.^12,20,23^

All patients with detectable metastases are classified as stage III regardless of the location and number of metastatic lesions; the prognostic relevance of the metastatic site at the time of diagnosis in SHSA has not yet been investigated in dogs. Understanding whether specific metastatic patterns are associated with distinct outcomes could refine prognostic stratification and support individualized treatment decisions.

Our aim was to describe the frequency and distribution of metastatic sites at the time of diagnosis in dogs with SHSA and assess their potential prognostic relevance.

## Materials and methods

### Study design and case selection

Medical records of dogs that underwent splenectomy with histologically confirmed SHSA between 2014 and 2024 from 3 different institutions were retrospectively examined.

The follow-up period was calculated from the day of surgery until tumor-related death or until last follow-up. Dogs were excluded if histologic confirmation was lacking or follow-up data were incomplete.

### Data collection

For each dog, data collected included breed, sex, age, body weight, clinical stage, and sites of metastasis at diagnosis and at the time of death if available, staging procedures performed, and histopathology results.

Dogs were staged based on World Health Organization (WHO) Guidelines^9^ (Table 1).

The diagnostic tests and imaging modality performed at the time of diagnosis were at the discretion of the attending clinician. Staging procedures included complete blood count (CBC), biochemistry, coagulation profile (prothrombin time [PT], activated partial thromboplastin time [aPTT], fibrinogen), and diagnostic imaging.

Diagnostic imaging techniques included abdominal ultrasonography (AUS) combined with 3-view thoracic radiographs performed either in awake or mechanically ventilated anesthetized patients and echocardiography or total body contrast-enhanced computed tomography (CT). Information related to staging results was obtained both from clinical records and imaging reports. Imaging studies were reviewed at the time of diagnosis and at every restaging by a board-certified radiologist as well as non-certified radiologists with > 10 years of experience in the field.

Patients were categorized by the clinician in charge of the case as stage III if single or multiple nodules in any target organ were identified by means of diagnostic imaging and if the number and appearance of the lesions were consistent with metastatic disease, as previously reported,^12,24–26^ or if the patient had a cytologic or histopathologic diagnosis of the lesion or lesions consistent with metastatic disease.

### Definition of clinical variables

Patients were considered anemic or thrombocytopenic if hemoglobin concentration or platelet numbers were below the reference intervals.

Two or more of the following abnormalities were used to define the presence of disseminated intravascular coagulation (DIC): thrombocytopenia, prolonged aPTT/PT or thrombin clot time, hypofibrinogenemia, decreased antithrombin activity (AT), increased markers of fibrinolysis (eg, fibrinogen degradation products, D-dimers), or erythrocyte fragmentation on a blood smear (eg, schistocytes, keratocytes, acanthocytes).^27^

#### Treatment

After surgery, owners were offered adjuvant treatment for their dogs with AC or MC-based protocols or a combination of both. Type of adjuvant treatment, such as AC or MC was recorded. The type of chemotherapy used included doxorubicin (DOX), epirubicin (EPI), and mitoxantrone (MTX). Doxorubicin and EPI were administered at a dosage of 30 mg/m^2^ or 1 mg/kg for dogs weighing <10-5 kg every 3 weeks as an IV infusion, whereas MTX was administered at a dosage of 5 mg/m^2^ as an IV infusion, with a 20% dose reduction for dogs weighing <10 kg, every 3 weeks. The intended number of AC doses was 5. Orally administered cyclophosphamide and chlorambucil were used in a MC regimen at a dosage of 10-15 mg/m^2^ PO once daily or every other day and 4 mg/m^2^ PO once daily, respectively, alone or in combination with thalidomide. Thalidomide dosage varied based on clinician preference and ranged between 2 and 8.7 mg/kg PO once daily in the evening. Chemotherapy adverse events were identified from the clinical records and graded according to the Veterinary Cooperative Oncology Group terminology criteria (VCOG-CTCAE, version 1.0 and 1.1).^28^

#### Response assessment

Dogs were restaged at the time of the fourth or fifth AC treatment or at the end of the protocol and then every 2-3 months, if no signs of disease progression were identified earlier. Dogs receiving MC usually were restaged every 2-3 months.

Procedures performed at the time of follow-up included hematology, biochemistry, 3-view thoracic radiographs combined with AUS or total body CT, depending on attending clinician and owner preference. Response to treatment was determined by the primary clinician’s assessment, according to the Veterinary Cooperative Oncology Group Response Evaluation Criteria in Solid Tumors (VCOG RECIST, version 1.0).^29^

Clinical follow-up was recorded at the time of the next reevaluation. Follow-up information not available in the medical records was obtained by telephone calls to the referring veterinarians, owners, or both.

### Statistical analysis

Descriptive and comparative statistics were performed. Descriptive statistics were reported for variables of breed, sex, age, and body weight.

Tumor specific survival (TSS) was used as an outcome measure and was defined as the time in days from splenectomy until tumor-related death. Dogs that were still alive at the end of the study, that had been lost to follow-up, or died of causes unrelated to SHSA, were censored.

Tumor specifi survival was calculated using the Kaplan–Meier product-limit method and the log-rank test to compare survival. Cases were censored if the dog was still alive at the end of the study, if the patient was lost to follow-up, or died of causes unrelated to SHSA.

Cox proportional hazard regression was used for univariate analysis of the continuous variables and for multivariate analysis. Variables were included in the multivariate analysis only if significant in the univariate analysis. For each test, differences were considered significant when *P* ≤ .05. Statistical analysis was performed using R (version 4.1.2 for Windows, Vienna, Austria).

### Cell line validation statement

No cell line validation was performed for this study.

## Results

### Study population

Sixty-six dogs were included in the study. There were 26 (39%) intact males, 9 (14%) neutered males, 25 (38%) spayed females, and 6 (9%) intact females. Median age was 10 years (range, 6-14) and median body weight was 28.2 kg (range, 3.5-45 kg). Twenty-two (33%) dogs were crossbreed dogs, followed by Labrador retriever (8, 12%), German shepherd (7, 10%), boxer (6, 9%), cocker spaniel (3, 4%), golden retriever (2, 3%), French bouledogue (2, 3%), beagle (2, 3%), and one each of the following: Akita inu, American Staffordshire terrier, Australian shepherd, Bernese mountain dog, Bolognese, Corso, dachshund, English bulldog, Gordon setter, Nova Scotia duck tolling retriever, Doberman pinscher, Pomeranian, Scottish terrier, Siberian husky.

### Staging procedures

At the time of diagnosis, 32 dogs (48%) were staged with three view thoracic radiographs combined with AUS, 34 dogs (52%) with a total body CT scan.

Three dogs (5%) were classified as stage I, 35 (53%) as stage II and 28 (42%) as stage III. Within the latter group, 11 (39%) were staged by a combination of thoracic radiographs and AUS, and 17 (61%) by total body CT scan.

For stage III dogs, the most common metastatic site at diagnosis was the liver (19, 28% of the entire population), followed by peritoneum (8, 12%), lungs (6, 9%), musculature (5, 8%), lymph nodes (2, 3%, including retropharyngeal lymph node in 1 case and both mediastinal and sternal lymph node in the other case), and kidneys (2, 3%; Table 2). Muscular metastases were distributed as follow: 2 cases involving appendicular muscles, 1 epiaxial, 1 thoracic wall, and 1 with both epiaxial and appendicular involvement.

**Table 2:** Distribution of metastatic sites at diagnosis and at death/restaging in the 66 dogs diagnosed with SHSA.

**Metastatic site**	**At diagnosis (stage III, *n*)**	**At death or restaging (*n*, cumulative)**
**Liver**	19 (28%)	45 (68%)
**Peritoneum**	8 (12%)	15 (23%)
**Lungs**	6 (9%)	8 (12%)
**Muscles**	5 (8%)	5 (8%)
**Lymph Nodes**	2 (3%)	1 (2%)
**Kidneys**	2 (3%)	2 (3%)
**Heart**	–	2 (3%)

“At Diagnosis” column reflects metastases identified at the time of initial staging.

“At Death or Restaging” column reflects cumulative metastases, including those known at diagnosis and those identified at restaging.

At the time of diagnosis, 6 dogs in stage III had 2 concurrent metastatic sites, the most common combination being the liver and peritoneum (4 cases) followed by liver and lung, and liver and kidney, respectively. Four dogs had 3 concurrent metastatic sites, with lungs, liver and muscles in 2 cases; lungs, kidney, and muscles in 1 case; and lungs, peritoneum, and lymph node in 1 case.

The most common metastatic site at the time of death was liver, with 45 cases (68%), followed by peritoneum (15, 23%), lungs (8, 12%), muscles (5, 8%), heart and kidney (2 cases each, 3%), and lymph nodes (1, 2%, including sternal and mediastinal; Table 2). Sixteen dogs (24%) had 2 metastatic sites at the time of death, with liver and peritoneum being the most common combination (10 cases, 41%), followed by liver and lungs in 4 cases (25%). Two patients had 3 metastatic sites documented at the time of diagnosis (peritoneum, kidney, and muscles and peritoneum, lungs, and lymph nodes, respectively).

Thirty-four dogs (57%) were restaged at the time of death, 23 cases (38%) had been restaged before death, whereas in 3 cases (5%) the information was not available. The mean interval from restaging to death was 8.8 days (95% CI, 4.9-12.7; range, 0-59), with a median of 0 days.

Histopathologic diagnosis of metastatic disease was available in 9 cases (14%), including 7 cases of hepatic metastasis and 9 cases of peritoneal metastasis. In all other cases, the diagnosis of metastatic disease was presumptive based on diagnostic imaging results.

On CBC, 42 dogs (63%) had anemia and 10 (15%) had thrombocytopenia. A coagulation profile was available in 41 patients and 3 dogs (7%) were diagnosed with DIC.

### Treatment

Fifteen patients (23%) underwent splenectomy alone, 19 (29%) received adjuvant AC-based chemotherapy, 8 (12%) a combination of AC-based chemotherapy and MC and 24 (36%) MC alone.

In the AC-based group, 11 (58%) received DOX, 5 (27%) DOX followed by MTX (in three cases because of reaching the maximum cumulative dose associated with cardiac toxicity, and in two cases after the occurrence of adverse effects), one (5%) MTX alone, one (5%) EPI included in a multiagent VAC (vincristine, doxorubicin, cyclophosphamide) protocol and one (5%) DOX included in a VAC protocol.

In the AC-based chemotherapy combined with MC, 4 dogs (50%) received DOX combined with cyclophosphamide, 2 (25%) MTX combined with cyclophosphamide and 2 (25%) a combination of DOX, cyclophosophamide, and thalidomide. In the MC group, 12 dogs (50%) received cyclophosphamide, 6 (25%) a combination of cyclophosphamide and thalidomide, 2 (8%) chlorambucil, 2 (8%) chlorambucil combined with thalidomide, one (4%) cyclophosphamide followed by chlorambucil and one (4%) thalidomide alone.

### Patient outcomes

At the time of death, 45 dogs (68%) had hepatic metastasis and 44 (64%) died of tumor-related hemoabdomen.

Fifty-four dogs (82%) died of tumor-related causes, with hemoabdomen being most common (44 cases, 66%). Six patients (9%) died of causes unrelated to the tumor (cardiac failure associated with myxomatous mitral valve disease stage C and D, neurologic signs, or unknown causes). Six patients (9%) were still alive at the end of the study.

Median TSS of the entire population was 132 days (95% CI, 14-948); TSS was 173 days for stage I (95% CI, 173-948), 162 days for stage II (95% CI, 123-195), and 65 days for stage III (95% CI, 51-110), resulting in an overall significant difference (*P* = .02). Although statistical analysis for the stage I group was not performed because of small sample size, Bonferroni testing identified a significant difference between stage II and stage III patients (*P* = .03).

Tumor specific survival was significantly decreased in patients that received surgery alone compared with those that received surgery combined with chemotherapy (60 vs. 173 days respectively, *P* < .001).

**Figure 1:**
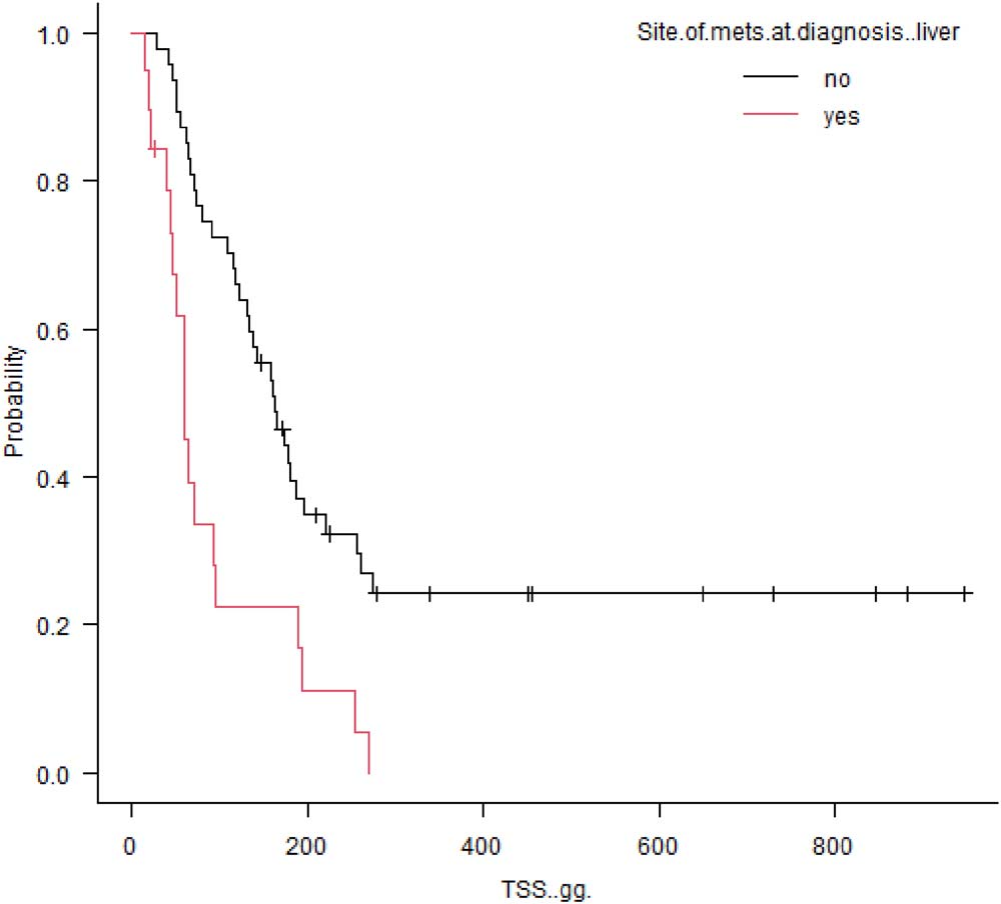
Kaplan-Meier survival curves comparing overall survival in 19 dogs with SHSA presenting with liver metastasis at diagnosis versus dogs without liver metastasis (TSS, 60 vs. 162 days, *P* = .0004). Vertical ticks represent censoring, and vertical continuous lines represent events.

**Figure 2:**
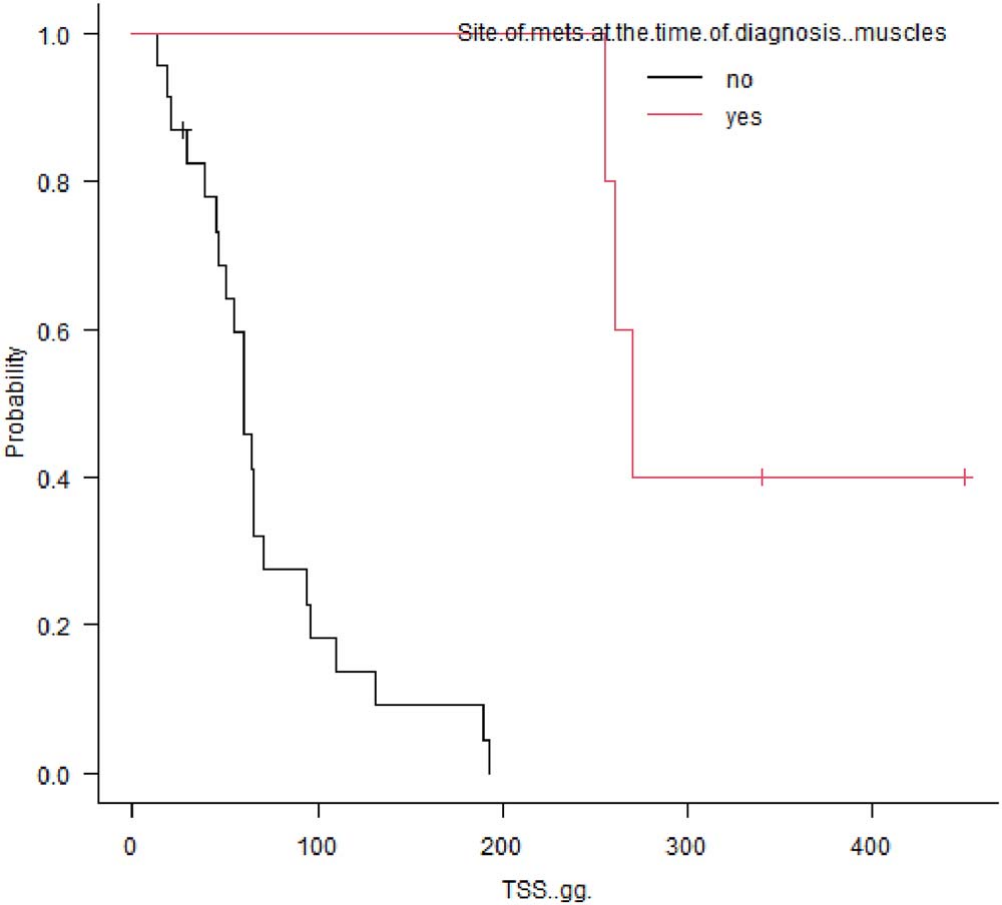
Kaplan-Meier survival curves comparing overall survival in 5 dogs with SHSA presenting with muscular metastasis at diagnosis versus dogs without muscular metastasis (TSS, 270 vs. 60 days; *P* = .0001).

**Figure 3:**
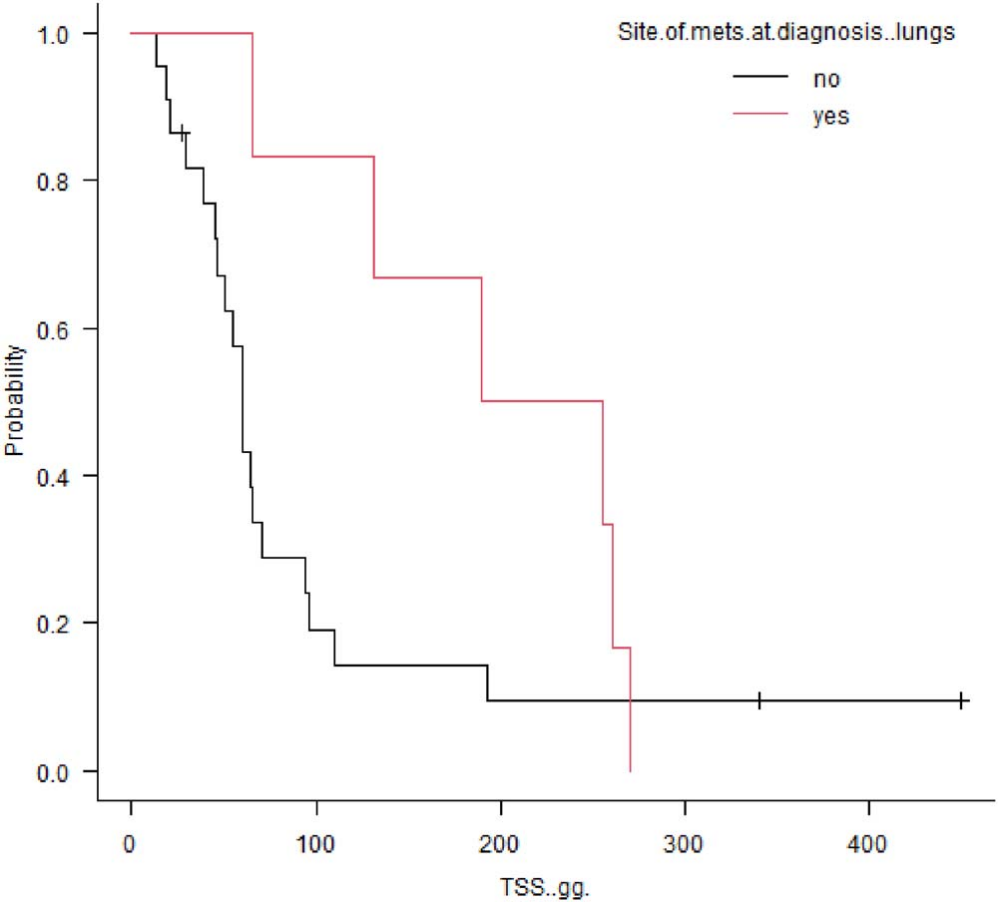
Kaplan-Meier survival curves comparing overall survival in 6 dogs with SHSA presenting with lung metastasis at diagnosis versus dogs without lung metastasis (TSS, 222.5 days vs. 60 days; *P* = .12).

Tumor specific survival was significantly decreased in patients with hepatic metastasis at diagnosis (60 vs. 162 days, *P* = .0004; Figure 1). Conversely, stage III patients with muscular metastasis had prolonged survival time compared with stage III patients without muscular metastasis (270 vs. 60 days; *P* = .0001; Figure 2). Also, among stage III patients, those with pulmonary metastasis had longer MST, but not significantly, compared with patients without pulmonary metastasis (222 days vs. 60 days; *P* = .121; Figure 3).

Considering the entire population regardless of clinical stage, the type of chemotherapy protocol (AC-based alone, AC-based combined with MC, MC alone) was not associated with a significant difference in TSS (133 vs. 170.5 vs. 195 days, respectively; *P* = .05). However, the type of chemotherapy used had a different impact on survival based on clinical stage. In fact, chemotherapy protocol in stage III patients was significantly associated with TSS, with patients that received an AC-based protocol having a better outcome compared with patients receiving MC (255 vs 65 days; *P* = .02).

Conversely, TSS was not influenced by the type of chemotherapy protocol in stage II patients (*P* = .05).

Hepatic metastases were negatively associated with TSS at the time of death, with a TSS of 90 days (95% CI, 64-138 days) compared with a TSS not reached for patients with metastasis to other sites (95% CI, 181-NA, *P* < .001).

On multivariate analysis, type of treatment (surgery vs. surgery and adjuvant chemotherapy) was an independent predictor of survival, with a hazard ratio (HR) of 0.174 and *P* < .001 (Table 3).

**Table 3:** Results of multivariable Cox proportional hazard regression analysis evaluating risk factors for death in dogs with SHSA.

	**Hazard ratio**	**Lower 95% CI**	**Upper 95% CI**	***P*-value**
**Stage II at diagnosis**	1.857	0.24920	13.8300	.55
**Stage III at diagnosis**	2.235	0.26990	18.5100	.46
**Type of treatment (surgery + chemotherapy)**	174	0.08326	0.3636	.000003
**Liver metastasis at diagnosis**	1.932	0.79450	4.6980	.15

## Discussion

Hemangiosarcoma is one of the most aggressive cancers in dogs, with tumor stage and treatment being the prognostic factors most commonly reported.^10,13–16^ In agreement with the current literature, our study confirms that stage at diagnosis and a multimodal approach combining surgery and chemotherapy positively correlate with survival, representing important prognostic factors for this tumor type.

At the time of diagnosis, metastases had occurred in approximately 42% of cases, with the liver being the most common site of metastases, as reported in previous studies.^4,5^ The liver also was the most common site at the time of death, and hemoabdomen was the cause of death in the majority of patients. On univariate analysis, hepatic metastasis negatively impacted TSS. This finding can be easily explained, considering that acute hemorrhage, especially in the presence of multiple lesions, cannot be managed with surgery, and chemotherapy is unlikely to have a therapeutic benefit.

Dogs with advanced clinical stage historically have had shorter survival times compared with dogs with early-stage disease^12–16^ but, surprisingly, the presence of muscular metastases was not associated with a worse outcome in our study population. The longer survival time in these patients could be explained by the fact that bleeding of lesions in this anatomical location might not affect patients as it does in the case of visceral locations, where bleeding is commonly associated with the development of fatal hemorrhage. Another explanation, as proposed in human medicine, is that muscles are a hostile environment for the retention and proliferation of cancer cells, because of muscle motion, muscle pH, and the ability of muscle to remove tumor-produced lactic acid.^30^

Recently, germline variants in somatic genes have been described in SHSA in dogs and, for some of them, including SETD2 and NOTCH1, an association with survival has been reported.^31^ Gene profiles in SHSA are not routinely evaluated in clinical practice, but gene profile might be an additional contributing factor to the different outcomes in SHSA patients.

Although not statistically significant, patients with pulmonary metastasis had longer survival in our study. A longer survival time for patients with pulmonary metastasis compared with hepatic metastasis has been described previously^4^: As reported by these authors, a possible explanation is that pulmonary metastasis are usually smaller and not cavitated and less prone to bleeding, compared with hepatic metastasis. Also, pulmonary nodules are generally smaller at the time of diagnosis and they tend to respond to treatment better than large, cavitated lesions in internal organs. In agreement with a previous study^32^ that observed different clusters of metastatic patterns in osteosarcomas in dogs, it is plausible that metastatic distribution reflects different biological behavior. Based on these findings, patients with muscular and pulmonary metastasis, that are classified as stage III based on the current staging system, could be considered as a separate entity with a separate prognosis compared with patients that have metastatic disease in other visceral sites.

Finally, evaluating the distribution of the different protocols in our population of stage III patients with lung metastasis, AC-based chemotherapy was used equally compared to MC protocols, suggesting that outcome was not strictly related to the use of dose intense chemotherapy, and other biological factors might explain the different outcome in these patients.

As previously mentioned, dogs received different treatments. All patients underwent splenectomy and the majority (78%) also underwent adjuvant chemotherapy, including different chemotherapy protocols. Patients that underwent surgery followed by adjuvant chemotherapy had longer survival than those treated with surgery alone, confirming previous findings.^5,7,19^ Regarding adjuvant treatment, several options have been described in the veterinary literature, including protocols based on administration of chemotherapy at the maximum tolerated dose or in a metronomic fashion or including antiangiogenic drugs, such as thalidomide.^33^

Considering the entire population, regardless of clinical stage, the type of chemotherapy protocol was not associated with a significant difference in overall MST, confirming previous findings,^11,22,24^ where the use of chemotherapy, regardless of the protocol used, was beneficial in these patients. However, when classifying patients based on clinical stage, AC-based chemotherapy resulted in a survival advantage in stage III patients, suggesting that MC might be less effective in the setting of macroscopic disease, as previously reported.^23^ However, a potential selection bias cannot be excluded, because dogs with advanced disease may have been preferentially selected to receive AC-based chemotherapy, which could have contributed to the observed survival advantage.

Our study had some limitations related to its retrospective nature, including the small number of patients included in the different subcategories of stage lowering statistical power; heterogeneity in staging, in the choice of adjuvant treatment, and in the type of chemotherapy protocols; and, the lack of a cytologic or histopathologic confirmation of metastasis in the majority of patients. Furthermore, potential selection biases related to treatment decisions across institutions should be acknowledged. Although all owners were offered comparable therapeutic options, clinical judgment may have influenced the choice of treatment based on disease extent at presentation. In particular, dogs with a lower burden of pulmonary metastases may have been considered suitable candidates for splenectomy more often than those with extensive metastasis or non-surgical abdominal metastases.

Patients were considered to have metastatic disease at the time of diagnosis and at the time of death mainly based on results of diagnostic imaging. Additionally, patients with surgically accessible lesions and low anesthesia risk underwent lesion sampling, regardless of macroscopic appearance. However, in higher anesthesia risk unstable patients or in cases with inaccessible lesions, these factors may have led to an underestimation of metastatic disease. The presence of multiple nodules in target organs or signs of hemoabdomen associated with multiple nodules in target abdominal organs were used to classify patients as having metastatic disease and to attribute a tumor-related cause to clinical signs. Although finding multiple nodules in dogs with a malignant neoplasm is suggestive of metastasis, other causes could not be ruled out without a histopathological diagnosis because necropsy examination was not routinely performed.

Another limitation was a non-standardization of the staging procedures, with 48% of patients undergoing thoracic radiographs and AUS, with the risk of not detecting lesions at other sites, such as muscular metastasis, only visible on CT scan. Advanced diagnostic imaging also was not performed in all patients during restaging and none of the patients had necropsy performed to confirm the cause of death.

In conclusion, tumor-related hemoabdomen was the most common cause of death in SHSA, with hepatic metastasis being associated with a worse prognosis compared to other metastatic sites. Patients with stage III disease and hepatic metastasis had better outcome when treated with AC-based chemotherapy protocols. Patients with pulmonary and muscular metastasis had a better prognosis compared with patients with hepatic metastasis. Additional studies on larger numbers of cases are needed to confirm our findings.

## Data Availability

The data that support the findings of this study are available on request from the corresponding author. The data are not publicly available due to privacy or ethical restrictions.

## Off-label antimicrobial declaration

Authors declare no off-label use of antimicrobials.

## Institutional animal care and use committee or other approval declaration

This retrospective study included client-owned dogs, with informed consent obtained at admission. Dog care was provided in accordance with good clinical practice standards. Ethical approval was not required by the lead author’s institution Clinica Veterinaria Malpensa AniCura for a retrospective study in which no research was conducted on live animals.

## Abbreviations

AC: anthracycline
AT: antithrombin
AUS: abdominal ultrasonography
CT: computed tomography
DIC: disseminated intravascular coagulation
DOX: doxorubicin
EPI: epirubicin
HR: hazard ratio
MC: metronomic chemotherapy
MTX: mitoxantrone
PT: prothrombin time
aPTT: activated partial thromboplastin time
SHSA: splenic hemangiosarcoma
TSS: tumor- specific survival
VAC: vincristine, doxorubicin, cyclophosphamide
VCOG: veterinary cooperative oncology group
